# Modulating the myosin super-relaxed state as a therapeutic strategy for myosin light chain–linked cardiomyopathies

**DOI:** 10.1007/s12551-026-01420-3

**Published:** 2026-02-09

**Authors:** Danuta Szczesna‐Cordary

**Affiliations:** Department of Molecular and Cellular Pharmacology, University of Miami Miller School of Medicine, Miami, FL 33136 USA

**Keywords:** Hypertrophic cardiomyopathy (HCM), Dilated cardiomyopathy (DCM), Restrictive cardiomyopathy (RCM), Myosin light chains, Super-relaxed (SRX) state, Disordered relaxed (DRX) state, MantATP displacement assay

## Abstract

In this review, we summarize a series of studies focused on cardiac myosin light chains and hereditary human mutations that cause hypertrophic (HCM), restrictive (RCM), or dilated (DCM) cardiomyopathy. In the heart, myosin serves as the molecular motor that converts the chemical energy of ATP hydrolysis into mechanical force, enabling cardiac contraction and blood pumping. Both myosin light chains, the regulatory (RLC) and essential (ELC), play critical roles in supporting and fine-tuning myosin motor function. Special emphasis is placed on the myosin super-relaxed (SRX) state, first described by Roger Cooke and colleagues more than 15 years ago. During diastole and muscle relaxation, myosin heads dynamically transition between two energetic states: the SRX state, which minimizes ATP consumption and preserves energy, and the disordered relaxed (DRX) state, in which myosin heads are more available for actin interaction but exhibit higher ATP turnover. To elucidate mechanisms underlying mutation-dependent pathological cardiac remodeling, we assessed the relative occupancy of myosin heads between the SRX and DRX states in skinned cardiac fibers from mouse models of HCM, RCM, and DCM using single-nucleotide turnover assays developed by the Cooke laboratory. Collectively, these studies demonstrate that mutation-induced alterations in myosin energetic states and dysregulation of the SRX:DRX balance constitute a central mechanism driving the distinct clinical and functional phenotypes observed in human cardiomyopathies caused by mutations in myosin RLC and ELC.

## Introduction

Pathological cardiac remodeling in hypertrophic (HCM), dilated (DCM), and restrictive (RCM) cardiomyopathies is predominantly caused by genetic mutations in thick- and thin-filament proteins, including β-myosin heavy chain (β-MHC), the myosin regulatory (RLC) and essential (ELC) light chains, myosin binding protein C (MyBP-C), titin, actin, tropomyosin (Tm), and the troponin (Tn) complex (TnT, TnI, and TnC) (Alcalai et al. 2008; Seidman and Seidman 2011). Amino acid substitutions or truncations in these proteins lead to severe consequences for cardiac muscle contraction, the ATP cycle, and the Ca^2+^/Tm/Tn-dependent regulation of contraction (Geeves and Holmes 2005; Lin et al. 2017).

HCM is an inherited, autosomal dominant disorder characterized by thickening of the left ventricular (LV) wall and interventricular septum (IVS), disorganized myofilaments, and interstitial fibrosis. Affecting approximately 1 in 200–500 individuals, HCM is a major contributor to sudden cardiac death (SCD) in young adults (Semsarian et al. 2015a, 2015b). RCM, a rarer and more severe variant of HCM, is defined by increased LV stiffness without accompanying wall thickening, leading to impaired diastolic filling and reduced cardiac output (CO) (Jean-Charles et al. 2011). Both HCM and RCM compromise the heart’s ability to fill and pump blood, ultimately resulting in diastolic dysfunction and reduced perfusion of peripheral organs (Olson et al. 2002). DCM, by contrast, is characterized by enlargement of the LV cavity, thinning or normal thickness of the LV wall, interstitial fibrosis, and a pronounced reduction in contractile performance (Halliday et al. 2017). DCM is one of the most prevalent forms of cardiomyopathy in the United States, with an estimated incidence of 1 in 400 individuals (Hershberger et al. 2013). While DCM may arise sporadically from ischemic injury, alcohol abuse, or viral infections, up to 25% cases have a genetic basis. Within the sarcomeric protein group, DCM has been linked to mutations in at least nine genes, including those encoding β-MHC, α-MHC, MyBP-C, titin, cardiac actin, the Tn complex, and α-Tm (Piran et al. 2012). Recently, the cardiac myosin RLC (*MYL2* gene) carrying the D94A mutation was implicated in causing dilated cardiomyopathy in patients (Huang et al. 2015).

Whereas pathological cardiomyopathies are marked by detrimental structural and functional changes, physiological cardiac hypertrophy represents an adaptive response characterized by increased heart size without abnormal morphology, preserved or even enhanced cardiac function (Gomes et al. 2015), a gene expression profile typical of healthy myocardium (Bernardo et al. 2010), and reversibility. Despite cardiac enlargement, physiological hypertrophy induced by exercise training is driven by cardioprotective signalling pathways and may help treat or prevent heart failure (McMullen and Jennings 2007).

### Myosin light chains: small proteins, big impact

As part of the heart’s molecular motor, the myosin cross-bridge hydrolyzes ATP and interacts with actin to generate force and movement. The lever arm translates subtle conformational changes in the motor domain, triggered by ATP hydrolysis, into larger structural shifts that result in sarcomere shortening and contraction (Rayment et al. 1993a, 1993b). The two light chains, RLC and ELC, are bound to their respective IQ (IQxxxRGxxxR) motifs within the myosin lever arm domain, where they stabilize its structure. Both RLC and ELC belong to the EF-hand calcium-binding protein family, which also includes calmodulin and TnC. The EF-hand superfamily encompasses a wide variety of Ca^2^⁺-regulated proteins with diverse cellular functions, all sharing a conserved helix-loop-helix fold consisting of a 12-amino acid Ca^2+^-binding loop flanked by two perpendicular α-helices (EF-hand motif) (Kretsinger 1976).

Growing evidence indicates that RLC and ELC modulate cardiac muscle contraction in a light-chain–specific manner. The localization of the RLC at the head-rod junction of the myosin molecule suggests a critical role in cross-bridge cycling, while its phosphorylation by Ca^2+^/calmodulin-activated myosin light chain kinase (MLCK) provides an additional level of regulation (reviewed in Szczesna-Cordary 2003; Yadav and Szczesna-Cordary 2017; Chang et al. 2015; Wang et al. 2014)). Myosin phosphorylation was a central and enduring theme in Roger Cooke’s research program, particularly in the earlier phases of his career. Several of his most cited papers from the late 1970 s and 1980 s examined the effects of phosphorylation on actin-myosin interactions, force generation, and energy utilization (Cooke et al. 1982; Franks et al. 1984). He and others postulated that phosphorylation decreases the ordering of myosin heads within the thick filament, shifting them into a more disordered state and thereby making them more “ready” to interact with actin (Ritz-Gold et al. 1980; Levine et al. 1995; Naber et al. 2011).

Interestingly, our lab recently demonstrated that mimicking myosin RLC phosphorylation in cardiac muscle alters contractility and improves overall myocardial performance in hearts carrying an HCM-associated RLC mutation (Yadav et al. 2019b). Notably, transgenic mice expressing an HCM mutation in combination with the S15D phosphomimetic substitution do not exhibit a pathological phenotype and do not develop hypertrophy (Yuan et al. 2015). Mutations in the ventricular RLC further underscore the importance of RLC phosphorylation, as they disrupt its phosphorylation status and alter the protein’s structural and functional properties (Muthu et al. 2012). Most RLC mutations are associated with HCM, with only a single variant, D94A, linked to DCM (Huang et al. 2015).

Similarly, mutations in the ventricular ELC (*MYL3* gene) have been implicated in HCM (reviewed in (Sitbon et al. 2020b)). These variants often affect regions essential for myosin structural stability and its interaction with actin, thereby altering cross-bridge kinetics and disrupting the fine regulation of cardiac muscle contraction (Lossie et al. 2012). A functionally important region of the cardiac ELC is its N-terminus (N-ELC), a rod-like, 91-Å-long extension that can bridge the ELC core of the myosin head to the actin filament (Aydt et al. 2007; Kazmierczak et al. 2009). The N-ELC plays a critical role in actin–myosin interactions, force generation, and muscle contraction under both physiological and pathological conditions (Wang et al. 2016, 2018; Yuan et al. 2017). To investigate its functional significance in vivo, we developed a transgenic mouse model of physiological-like hypertrophy expressing a 43-amino-acid N-terminal truncation of ELC (Δ43 mice) (Kazmierczak et al. 2009). These mice exhibited cardiac enlargement without pathological remodeling or functional impairment (Muthu et al. 2011).

It is important to note that although disease-causing mutations in both light chains are far less common than those in β-MHC or MyBP-C, they are often associated with severe, malignant clinical outcomes. This underscores the need for greater attention to RLC and ELC in inherited cardiomyopathies, as these small yet essential proteins exert disproportionately large effects on sarcomeric function, cardiac energetics, and disease progression (Yadav et al. 2019a).

### Modulation of the super-relaxed state by myosin light chain mutations

Biochemical and structural studies of sarcomeric proteins demonstrate that HCM-associated mutations lead to dysregulation of the myosin super-relaxed (SRX) state, a biochemical state of myosin first described by Roger Cooke in skeletal muscle (Stewart et al. 2010) and later characterized in cardiac muscle (Hooijman et al. 2011). The SRX state is characterized by markedly inhibited ATPase activity and, in cardiac muscle, is considered an energy-conserving configuration that provides a cardioprotective function in the working heart. In contrast, in the disordered relaxed (DRX) state, myosin heads extend into the interfilament space, where they are more readily available to bind actin and form cross-bridges, albeit at the cost of increased ATP consumption (Hooijman et al. 2011). Structurally, the SRX is thought to involve the formation of the interacting-heads motif (IHM), in which the two myosin heads interact asymmetrically and fold back against the myosin backbone (Dutta et al. 2023; Grinzato et al. 2023; Alamo et al. 2017). In the DRX state, by contrast, myosin heads project outward into the interfilament space and adopt a range of conformations, some of which are primed for actin binding and force generation (Jani et al. 2024). Structural studies further indicate that mutations that destabilize the SRX state also disrupt the IHM, a key structural configuration associated with maintenance of the SRX state (Grinzato et al. 2023). Such destabilization is thought to reduce energetic efficiency in the heart and contribute to pathological cardiac remodeling.

**Fig. 1:**
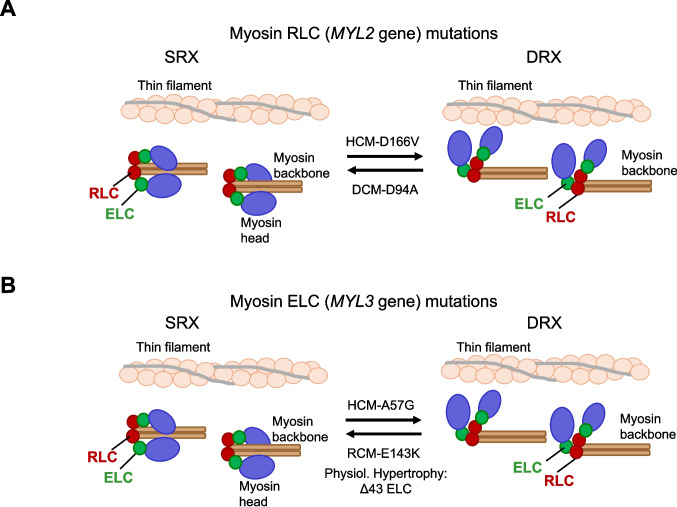
The effect of cardiomyopathy mutations in myosin RLC (**A**) and ELC (**B**) on the distribution of myosin cross-bridges between the super-relaxed (SRX) and disordered relaxed (DRX) states. The truncation mutation in myosin ELC (Δ43), which stabilizes the SRX state and induces cardiac hypertrophy in animals older than 7 months, is also shown

Inspired by Roger Cooke’s discovery, we sought to define mutation-dependent dysregulation of the SRX state in our mouse models of cardiomyopathy using a single-nucleotide turnover assay as described in (Hooijman et al. 2011; Jones et al. 2025). Our goal was to elucidate how the SRX:DRX balance is differentially regulated across distinct forms of cardiomyopathy. We found that HCM-associated mutations in both myosin RLC and ELC shift the relative occupancy of myosin heads from SRX-like toward DRX-like states, thereby increasing the number of myosin heads available for actin interaction (Fig. 1). These changes correlate with the hypercontractility observed in HCM patients and in HCM mouse models developed in our laboratory (Sitbon et al. 2024; Yuan et al. 2015, 2022) and by others (Pietsch et al. 2025; McNamara et al. 2017). It is important to note that while mantATP displacement assays consistently resolve two kinetic populations, recent work has highlighted that these phases may arise from structurally and kinetically distinct myosin sub-populations rather than a single rapidly equilibrating SRX–DRX pair (Mohran et al. 2024; Walklate et al. 2022).

### Mutations in myosin RLC

The question that we address in this section concerns the molecular basis for phenotypic differences between two RLC mutations: D166V (Aspartic acid to Valine), which causes HCM (Richard et al. 2003; Correction 2004), and D94A (Aspartic acid to Alanine), identified by the Hershberger group as causing DCM (Huang et al. 2015). Skinned papillary muscles from hypertrophic (HCM-D166V) and dilated (DCM-D94A) cardiomyopathy mouse models were analyzed using small-angle X-ray diffraction in combination with isometric force measurements (Yuan et al. 2022). This approach allowed us to determine interfilament lattice spacing, equatorial intensity ratios (I₁₁/I₁₀), and the force-pCa relationship across the full range of calcium concentrations at a sarcomere length of 2.1 μm. In parallel, we evaluated how these mutations affected ATP-dependent myosin energetic states and the SRX/DRX ratio.

Compared with wild-type (WT) RLC and DCM-D94A mice, HCM-D166V significantly increased Ca^2+^ sensitivity of force and induced a leftward shift in the I₁₁/I₁₀—pCa relationship, consistent with myosin heads moving closer to actin-containing thin filaments and thereby enabling premature Ca^2+^ activation. Importantly, HCM-D166V also disrupted the SRX state and promoted a transition to the DRX state, correlating with the hypercontractile phenotype characteristic of HCM (Fig. 1A). This dysregulation of the SRX state and the shift toward DRX is consistent with the observed repositioning of myosin cross-bridges toward the thin filament and the enhanced force-pCa sensitivity in HCM-D166V hearts (Kerrick et al. 2009).

In contrast, the DCM-D94A mutation favored the energy-conserving SRX state, with structural I₁₁/I₁₀—pCa parameters that largely resembled WT. The increased proportion of myosin heads residing in the SRX state in DCM-D94A mice, accompanied by reduced ATP consumption (Yuan et al. 2022), is consistent with our earlier findings showing D94A-induced reductions in actin-activated myosin ATPase activity and the low ejection fraction observed in D94A mice in vivo (Yuan et al. 2018). The altered SRX regulation observed across our animal models of RLC-related cardiomyopathy provides a unifying framework for understanding the development of hypercontractile versus hypocontractile phenotypes, each accompanied by substantial changes in metabolic demand (Fig. 1A).

### Mutations in myosin ELC

In this section, we examine the molecular basis underlying the distinct phenotypes produced by two myosin ELC mutations: the HCM-associated A57G (Alanine to Glycine) and the RCM-associated E143K (Glutamic acid to Lysine) variants (Lee et al. 2001; Choi et al. 2010; Olson et al. 2002; Caleshu et al. 2011).

In transgenic mice expressing the A57G mutation in the heart, LV tissue displayed hallmark features of hypertrophic cardiomyopathy, including increased myofilament Ca^2^⁺ sensitivity of force, elevated stroke work (SW), augmented CO, and significant fibrosis (Kazmierczak et al. 2013; Muthu et al. 2011). We also assessed the impact of A57G on the myosin SRX state (Sitbon et al. 2020a), a key regulator of sarcomere function extensively characterized by the Cooke team and colleagues (Hooijman et al. 2011; Toepfer et al. 2020; Anderson et al. 2018; McNamara et al. 2015). A57G fibers exhibited a shift from the SRX to the DRX state, likely contributing to hypercontractile myosin behavior and the pathological remodeling seen in A57G hearts (Sitbon et al. 2020a) (Fig. 1B).

In transgenic mice expressing the E143K mutation, we observed a phenotype that closely parallels human restrictive cardiomyopathy, characterized by impaired diastolic filling, reduced SW and CO, myocardial fibrosis, and a modest increase in myofilament Ca^2^⁺ sensitivity of force generation in male mice (Yuan et al. 2017). Remarkably, Sitbon et al. (Sitbon et al. 2021) showed that E143K fibers favored the SRX state, suggesting fewer myosin cross-bridges available for contraction (Fig. 1B). This shift in myosin head occupancy toward the SRX state provides a mechanistic explanation for myosin’s hypocontractile activity and the compromised cardiac performance previously documented in E143K mice (Yuan et al. 2017).

Given these ELC mutation–specific alterations in myosin energetic states, we next asked whether deletion of the N-ELC in HCM-A57G or RCM-E143K mice could serve as a therapeutic strategy to mitigate the pathological phenotypes caused by these disease-associated mutations. It is important to note that transgenic Δ43 mice older than seven months develop substantial cardiac hypertrophy but exhibit no detectable histopathology or fibrosis (Muthu et al. 2011). Moreover, Δ43 mice were shown to stabilize the low-energy SRX state (Sitbon et al. 2020a), a pattern also observed in RCM-E143K fibers (Sitbon et al. 2021) and in DCM-D94A RLC mice (Yuan et al. 2022) (Fig. 1B). Our studies on double-mutant mice revealed the following: 1) Partial N-ELC ablation through introduction of the Δ43 variant in A57G × Δ43 double-mutant mice effectively reversed the cardiac hypertrophy and fibrosis characteristic of HCM-mutant hearts, restored key parameters of cardiac function, and normalized the disrupted SRX state (Sitbon et al. 2024). 2) In contrast, E143K × Δ43 double-mutant mice showed no improvement in cardiac structure or function relative to the RCM-mutant alone, and fibrotic remodeling persisted (Sitbon et al. 2024). These findings provide important insights into the essential role of the cardiac myosin ELC and its N-terminus in regulating myosin motor function, as well as the complex mechanisms underlying cardiomyopathies (Sitbon et al. 2024).

## Final remarks

Building on Roger Cooke’s visionary discovery of the myosin super-relaxed state, our studies using mouse models of cardiomyopathy and physiological-like hypertrophy demonstrate that mutation-induced shifts in myosin energetic states are fundamental determinants of cardiac phenotype. Alterations in the relative occupancy between the energy-conserving SRX state and the more active disordered relaxed (DRX) state emerge as a central mechanism linking sarcomeric mutations in myosin light chains to divergent patterns of cardiac remodeling and dysfunction. Dysregulation of the SRX–DRX balance modulates myosin head availability, ATP consumption, and cross-bridge recruitment, thereby influencing contractile performance, energetic efficiency, and disease progression.

Consistent with this framework, HCM-associated D166V and A57G mutations in myosin RLC and ELC, respectively, destabilize the SRX state and promote a shift toward the DRX state, correlating with the hypercontractile phenotype characteristic of HCM (Fig. 1). Destabilization of the SRX state may impair cardiac energetic efficiency and contribute to the development of pathological remodeling. In contrast, the DCM-associated D94A mutation in myosin RLC favors stabilization of the energy-conserving SRX state, accompanied by reduced ATP consumption and an increased proportion of myosin heads residing in the SRX state. Similarly, the physiological-like hypertrophy Δ43 ELC model and the RCM-associated E143K mutation in myosin ELC stabilize the SRX state, suggesting reduced availability of myosin cross-bridges for force generation.

Collectively, these findings provide a unifying mechanistic framework for understanding how mutations in myosin RLC and ELC give rise to the distinct clinical and functional manifestations of hypertrophic, restrictive, and dilated cardiomyopathies, and they highlight modulation of myosin energetic states along the SRX–DRX axis as a promising target for future therapeutic intervention.

## Data Availability

No datasets were generated or analysed during the current study.
